# Comparison of the Kinetic Parameters of Alternative Oxidases From *Trypanosoma brucei* and *Arabidopsis thaliana*—A Tale of Two Cavities

**DOI:** 10.3389/fpls.2021.744218

**Published:** 2021-10-22

**Authors:** Fei Xu, Alice C. Copsey, Luke Young, Mario R. O. Barsottini, Mary S. Albury, Anthony L. Moore

**Affiliations:** Biochemistry and Biomedicine, School of Life Sciences, University of Sussex, Brighton, United Kingdom

**Keywords:** alternative oxidase, AOX inhibitors, oxygen and quinol kinetics, quinol oxidation, respiration, activators

## Abstract

The alternative oxidase (AOX) is widespread in plants, fungi, and some protozoa. While the general structure of the AOX remains consistent, its overall activity, sources of kinetic activation and their sensitivity to inhibitors varies between species. In this study, the recombinant *Trypanosoma brucei* AOX (rTAO) and *Arabidopsis thaliana* AOX1A (rAtAOX1A) were expressed in the *Escherichia coli* Δ*hemA* mutant FN102, and the kinetic parameters of purified AOXs were compared. Results showed that rTAO possessed the highest *V*_max_ and *K*_m_ for quinol-1, while much lower *V*_max_ and *K*_m_ were observed in the rAtAOX1A. The catalytic efficiency (*k*_cat_/*K*_m_) of rTAO was higher than that of rAtAOX1A. The rTAO also displayed a higher oxygen affinity compared to rAtAOX1A. It should be noted that rAtAOX1a was sensitive to α-keto acids while rTAO was not. Nevertheless, only pyruvate and glyoxylate can fully activate Arabidopsis AOX. In addition, rTAO and rAtAOX1A showed different sensitivity to AOX inhibitors, with ascofuranone (AF) being the best inhibitor against rTAO, while colletochlorin B (CB) appeared to be the most effective inhibitor against rAtAOX1A. Octylgallate (OG) and salicylhydroxamic acid (SHAM) are less effective than the other inhibitors against protist and plant AOX. A Caver analysis indicated that the rTAO and rAtAOX1A differ with respect to the mixture of polar residues lining the hydrophobic cavity, which may account for the observed difference in kinetic and inhibitor sensitivities. The data obtained in this study are not only beneficial for our understanding of the variation in the kinetics of AOX within protozoa and plants but also contribute to the guidance for the future development of phytopathogenic fungicides.

## Introduction

The alternative oxidase (AOX) is a terminal oxidase that introduces a branch point in the mitochondrial electron transport chain (ETC) at the ubiquinone pool (Moore and Siedow, 1991). The AOX catalyzes the oxidation of ubiquinol while reducing O_2_ to H_2_O, but unlike cytochrome c oxidase (complex IV), it does not translocate protons from the matrix to the intermembrane space (Vanlerberghe and McIntosh, 1997; Moore and Albury, 2008; Moore et al., 2013). This lack of proton translocation makes the alternative pathway less energy efficient, with less ATP being generated per molecule of glucose when compared to the regular ETC and the excess energy being dissipated as heat instead. The AOX is remarkably insensitive to inhibitors of the ETC, such as cyanide, nitric oxide, azide, and sulfide (McDonald, 2008; Moore et al., 2013).

The discovery and first characterization of a plant *AOX* gene occurred in the thermogenic arum, *Sauromatum guttatum* (Rhoads and McIntosh, 1991), where the protein is found in high concentrations, and its heat-generating properties are used to volatilize primary amines for pollination purposes (Skubatz et al., 1991). Since then, AOX activity analyses have been carried out in more plants, such as *Arum maculatum* (Leach et al., 1996), *Solanum lycopersicum* (Holtzapffel et al., 2003), and *Arabidopsis thaliana* (Umbach et al., 2002). As a commonly used model plant, there are five AOX isoform proteins in *A. thaliana*, but the AtAOX1A is thought to be more highly expressed in all tissues and plays a more fundamental role in the regulation of energy metabolism, where it is involved in facilitating tricarboxylic acid (TCA) cycle turnover, protection against oxidative stress, and the preservation of plant growth homeostasis (Crichton et al., 2010; Zhang et al., 2016; Selinski et al., 2017). In addition to plants, a broad distribution of the AOX has been found in protists, fungi, and microsporidian parasites (McDonald, 2008; Williams et al., 2010). Notably, the human parasite *Trypanosoma brucei* contains an AOX (TAO), and cellular respiration measurement of the bloodstream form has confirmed that it solely relies on TAO (Clarkson et al., 1989; Chaudhuri et al., 2006). It is assumed that the AOX occurs in prokaryotes and enters the eukaryotic cell lineage through major symbiotic events, but AOX activity is quite different in prokaryotes and eukaryotes and is regulated by different effectors (McDonald, 2008; Moore and Albury, 2008).

As mentioned above, AOXs from different organisms present different biochemical features regardless of the fact that the enzymatic active site is virtually conserved in all AOXs identified to date. For instance, plant AOXs are activated by organic acids (Millar et al., 1993; Umbach et al., 2002; Selinski et al., 2018). In contrast, the AOX in fungi and protists is generally activated by mono- and diphosphate nucleosides, such as adenosine monophosphate (AMP), guanosine monophosphate (GMP), and adenosine diphosphate (ADP) (Jarmuszkiewicz et al., 2005; Barsottini et al., 2020). Those allosteric activation sites have been traced to unique features in their primary structure, such as an exclusively conserved cysteine in plant AOXs (Umbach and Siedow, 2000; Umbach et al., 2006). In addition, it has been speculated that the vast variation in catalytic efficiency between AOXs is caused not by differences in the substrate binding site, but in the entrance of the hydrophobic tunnel that leads to the binding site (May et al., 2017). However, there is still a considerable amount of research to be carried out in order to reveal the mechanism of activation of AOXs.

In the past few decades, the AOX has been regarded as a functional marker for the breeding of plants with increased resistance to stress, and it is also a candidate for gene therapy to treat mitochondrial dysfunction diseases in humans (Van Aken et al., 2009; Costa et al., 2014). Moreover, chemical AOX inhibitors have potential applications in the control of human parasites and plant pathogens (Mallo et al., 2014; West et al., 2018; Duvenage et al., 2019). It is, therefore, clear that the study of AOXs and the elucidation of structure-activity relationships can provide insights about this family of proteins and be useful for the development of new technologies. Therefore, the aim of this work is to characterize the AOX activities from two distinct eukaryotes, one from the protozoa *T. brucei* and the other from the model plant *A. thaliana*, and compare their kinetic and inhibitor-sensitive characteristics when the respective AOX is expressed in the same system. Particular attention was paid to ubiquinol and oxygen kinetics in the presence and absence of allosteric activators, and also to the effect of novel AOX inhibitors such as ascofuranone and its derivatives.

## Materials and Methods

### AOX Sequence Alignment and Structural Modeling

The amino acid sequences of the AOXs used here are from *T. brucei* (TAO; accession: BAB72245.1), and *A. thaliana* (AtAOX1A; accession: NP_188876.1). The sequence alignment was performed with DNAMAN (Lynnon Corporation, version 9.0.1.116) with default parameters. The homology modeling of the AtAOX1A protein was carried out using SWISS-MODEL (https://swissmodel.expasy.org/) based on the x-ray structures of TAO (PDB ID 3vv9 and 3w54). The protein structure alignment was performed using PyMOL 2.3.3 (Schrödinger, LLC), and the hydrophobic tunnels of the catalytic active center were analyzed using the Caver 3.0 software.

### Establishment of Overproduction System for rTAO and rAtAOX1A in *E. coli*

The establishment of the overproduction system for rTAO and rAtAOX1A in *E. coli* (FN102 heme-deficient strain) was performed according to the method of Nihei et al. (2003). The FN102 strain is auxotrophic for 5-aminolevulinic acid (ALA) as it lacks the glutamyl-tRNA reductase (gene hemA) required for the production of ALA, which is the first step in *E*. coli heme biosynthesis. The presence of ALA in the media is therefore crucial for the synthesis of active cytochrome *bd* quinol oxidase in the initial growth phase. Following AOX expression, the cytochrome *bd* oxidase becomes redundant, and ALA is removed from the bulk media to encourage further AOX expression. The rTAO and rAtAOX1A lacking the mitochondrial signal sequences were used for expression in the FN102 strain. The mature AOX sequences were removed on a *Nde*I-*Bam*HI fragment and ligated to *Nde*I-*Bam*HI digested pET15b, in which the 6 × His tag was replaced by a Twin-Strep tag to produce the expression construct pET.rTAO/rAtAOX1A.

### Membrane Preparation

Both the freshly inoculated culture and the starter culture contained 50 μg ml^−1^ ALA before the strain FN102/pAOXs was transferred to the fermentation growth. The strain FN102/pAOXs were grown aerobically at 30°C in 5 L K-broth (50 g tryptone–peptone, 25 g yeast extract, 25 g casamino acid, 52 g dipotassium hydrogen orthophosphate, 15 g potassium dihydrogen orthophosphate, 3.7 g trisodium citrate, 12.5 g ammonium sulfate, 0.25 g magnesium sulfate, 0.125 g iron sulfate, 0.125 g iron chloride, 10 g glucose, and 0.5 g carbenicillin). The cultures were incubated by shaking at 30°C (temperature reduced from 37°C to prevent the formation of inclusion bodies) until the OD_600_ = 0.6, at which point the cells were induced with 25 μM isopropyl β-d-1-thiogalactopyranoside (IPTG). Following induction, the cultures were incubated for a further 14 h at 30°C by shaking.

The cells were harvested after 14 h of culture and resuspended in 65 mM 3-(N-morpholino)propanesulfonic acid (MOPS, pH 7.5). After the pellets were pooled and homogenized, a protease inhibitor cocktail (Roche “complete”) was added, before lysis using a French Press (Thermo Electron; 30 k psi, two passes using SLM Aminco FA-078 press with an FA-032 cell). After lysis, cell debris was removed in a single 12,000 g centrifugation step, and the supernatant was centrifuged for 80 min at 200,000 g. The membrane pellet was resuspended in 65 mM MOPS (pH 7.5) and used as the membrane sample.

### Solubilization and Purification of rTAO and rAtAOX1A

Membranes containing rTAO and rAtAOX1A were incubated with a solubilization buffer (200 mM of MgSO_4_, 10% glycerol, and 65 mM of MOPS, pH 7.5) plus different detergents [1% (w/v) octyl-glucoside (OG) for rTAO; 1% (w/v) n-dodecyl-β-D maltopyranoside (DDM) for rAtAOX1A]. Following 1 h of solubilization at 4°C, samples were centrifuged at 200,000 g for 30 min.

For protein purification, solubilized proteins were applied to a twin-strep column (10 ml). The column was washed with water, then equilibrated with a washing buffer [65 mM MOPS, 0.05% DDM, 50 mM MgSO_4_, 150 mM NaCl, and 20% (v/v) glycerol, pH 7.5]. After the resin-bound AOX was transferred to a column, an elution buffer (washing buffer plus 2.5 mM of desthiobiotin) was used to elute the AOX. Purified proteins were stored at −80°C or used to do the assay as described in the following methods.

### Kinetics Measurement-Quinol

Kinetic analysis of the purified rTAO and rAtAOX1A was performed spectrophotometrically using a Cary 4000 UV spectrophotometer (Varian, UK Ltd, Surrey UK) with the packaged software. All assays were performed in 65 mM MOPS buffer pH 7.5 with different concentrations of quinol-1 as the substrate, measuring the conversion of Q_1_H_2_ (ubiquinol-1) to Q_1_ (ubiquinone-1) at 278 nm (ε = 15,000 M^−1^ cm^−1^). Q_1_ was reduced to Q_1_H_2_ prior to experimentation *via* sonication with zinc powder under an inert atmosphere for ~10 min, with the resultant zinc then being removed *via* centrifugation at 2,000 g.

### Kinetics Measurement-Oxygen

Oxygen consumption rates were measured with an Oxygraph-2k chamber (O2k; Oroboros Oxygraph-2k, Innsbruck, Austria) at a constant temperature of 25°C. The *E*. coli membrane fractions containing rTAO and rAtAOX1A were added to 2 ml of 65 mM MOPS (pH 7.5) with 1 mM KCN and the reaction was initiated with the addition of 1.25 mM NADH. In the present study, the O_2_ concentrations decreased from 100~120 to 0.5~1 nmol/ml and were used for the calculation of *K*_m_ for O_2_. In addition, we found that an oxygen consumption rate of ~1 nmol O_2_ min^−1^ mg^−1^ was sufficient to gather enough data points to be collected for the determination of O_2_ *K*_m_ using an Eadie–Hofstee graph.

### Activation Assay by Different Effectors

To determine whether the different effectors affect the oxygen consumption rate of rTAO and rAtAOX1A, succinate, pyruvate, glyoxylate, oxaloacetate (OAA), AMP, GMP, and GDP were added to the reaction chamber. The final concentration of each effector used in this study was 10 mM according to the titration results.

### IC_50_ Assay by Different Inhibitors

The inhibiting concentration of 50% (IC_50_) for the membrane-bound proteins of rTAO and rAtAOX1A were determined in a Microplate Spectrophotometer (Thermo Scientific™, Waltham, MA, USA) with inhibitors including ascofuranone (AF), ascochlorin (AC), colletochlorin B (CB), colletochlorin D (CD), octylgallate (OG), and salicylic hydroxamic acid (SHAM). For the IC_50_ measurement, absorbance changes of NADH at 340 nm with different inhibitor concentrations in a MOPS buffer (pH 7.5) were detected with 300 μM NADH as the substrate.

### Western Blot Analysis

Proteins were separated (at 120 V) on 12% SDS-polyacrylamide gels and transferred (at 20 V) to nitrocellulose membranes (0.45-mm pore size) in a buffer containing glycine (190 mM), methanol (10% v/v), and Tris (13 mM, pH adjusted to 7.2 with HCl) based on methods described by Affourtit and Moore (2004). The filters were incubated overnight at 4°C in 3% (w/v) bovine serum albumin (BSA) and 2% (w/v) milk powder in Tris-buffered saline (140 mM NaCl and 20 mM Tris, pH adjusted to 7.6 with HCl) supplemented with 0.1% (v/v) Tween-20 (TBST). Filters were washed in TBST and incubated for 1 h at room temperature with 2% (w/v) milk powder in TBST containing monoclonal antibodies (1:20,000 dilution) raised against a Twin-Strep tag. Following a wash in TBST, filters were detected using ECL™ Western Blotting Detection Reagents GE Healthcare (Sigma-Aldrich, St. Louis, MO, USA).

### Protein Content Assay

The content of rTAO and rAtAOX1A protein (including membrane protein and purified protein) was estimated using the Bradford method (Bradford, 1976), and the Bio-Rad protein assay dye used BSA as the standard. The signal intensity of immunodecorated rTAO membrane and rAtAOX1A membrane samples was measured by the Image J software, and the relative protein concentrations were calculated based on the purified rTAO and rAtAOX1A.

### Statistical Analysis

Statistical evaluations were conducted by means of a one-way ANOVA with a *post-hoc* Tukey HSD test integrated into GraphPad Prism 7 (GraphPad Software Inc.). Differences with *p* < 0.05 were considered as significant and indicated as an asterisk (^*^) or different letters.

## Results

### Recombinant AOX Proteins Expression and Purification

**Figure 1:**
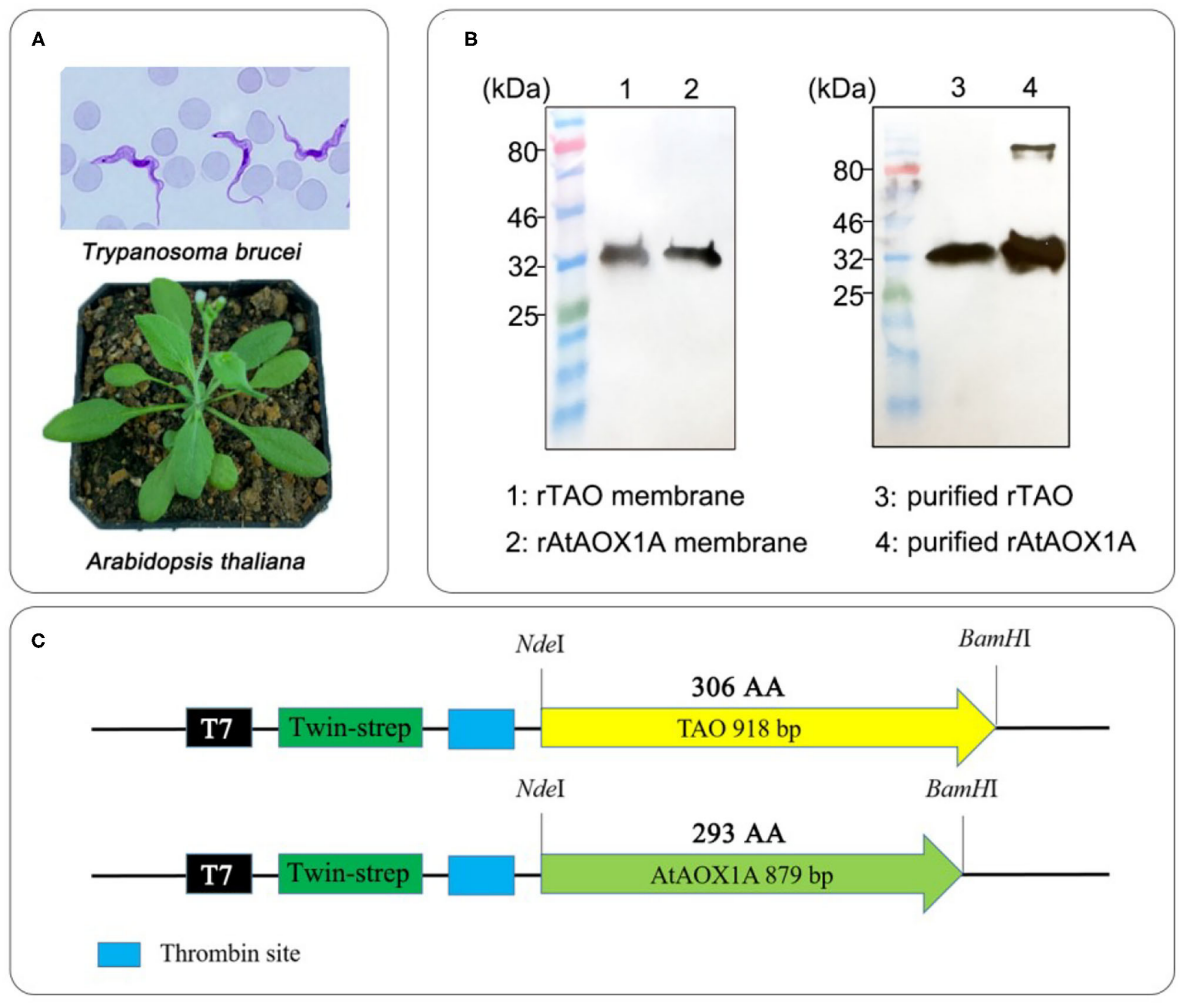
Alternative oxidase (AOX) from *Trypanosoma brucei* and *Arabidopsis thaliana*. **(A)** Phenotypes of *Trypanosoma brucei* (from CDC) and *A. thaliana*. **(B)** Western blot results for membrane-bound proteins and purified AOXs. Within this experiment, 15 μg of total membrane proteins and 1 μg purified proteins were used for the Western blot. **(C)** Schematic diagram of recombinant expression vector construction for AOXs.

In this study, rTAO and rAtAOX1A lacking the mitochondrial targeting signal sequence and fused to a twin-strep tag were expressed in the *E. coli* FN102 heme-deficient strain. The membrane-bound protein fraction was harvested and used for the further purification of the AOX protein with the aid of a Strep-Tactin chromatography resin. Protein samples before and after purification were compared by Western blot (Figure 1). A single band was observed in all three of the membrane-bound protein fractions, whereas some aggregation was observed in the purified rAtAOX1A, which was not observed for the purified rTAO. This may indicate the formation of dimers or polymers of the purified rAtAOX1A during purification.

### Comparison of the AOX Proteins Sequence and Homology Protein Structures

**Figure 2:**
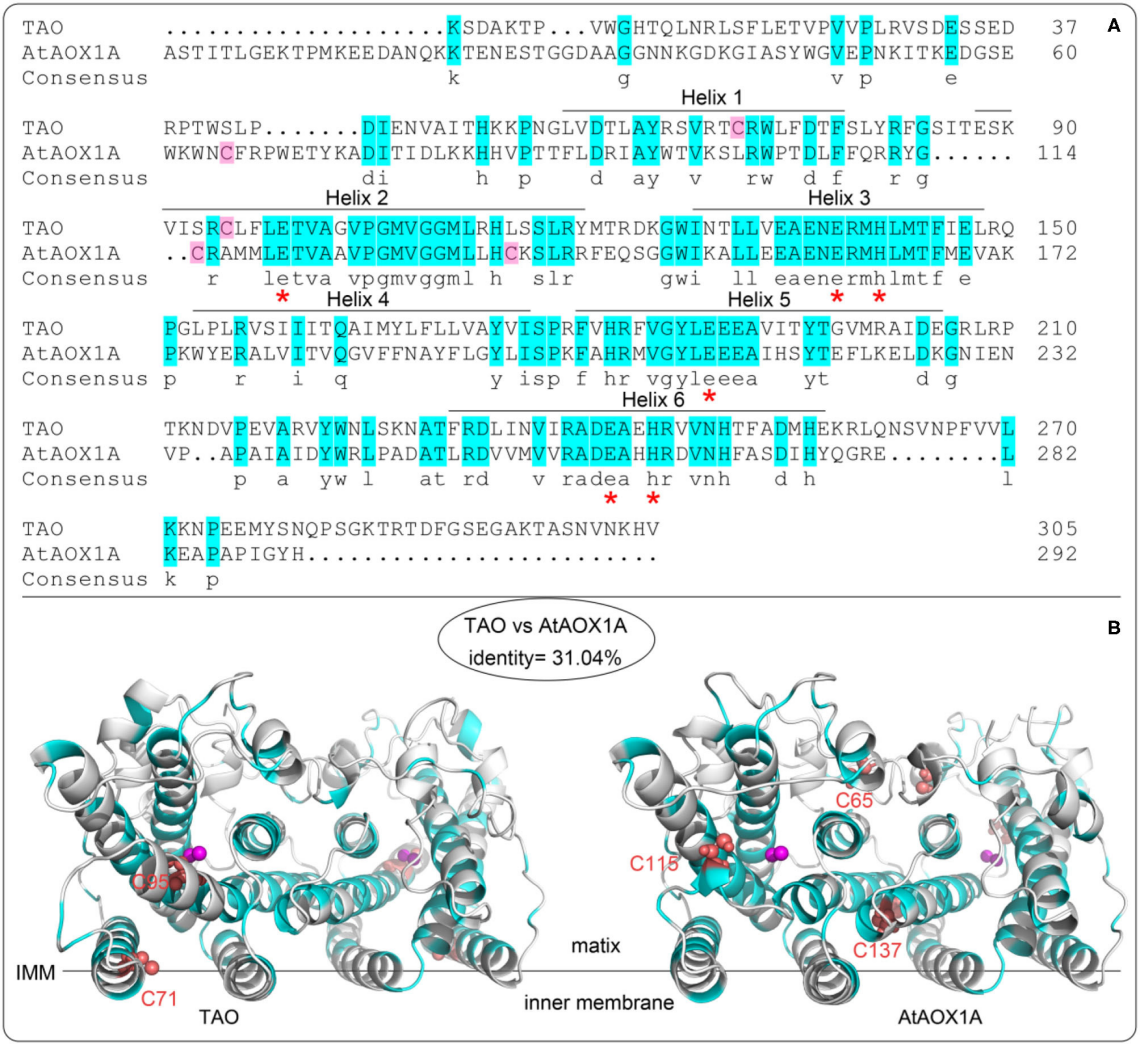
Alignment of protein sequences and homology structure of TAO and AtAOX1A. **(A)** Aminol acid sequence alignments between TAO and AtAOX1A without leading sequences. The blue background shows similar amino acids between TAO and AtAOX1A. Asterisks (red) represent the conserved amino acid residues binding to di-iron ferroxidase centers. The pink background shows the cysteine contained in AOXs. **(B)** Comparison of cysteine sites based on the secondary structure of TAO and AtAOX1A. Purple sphere shows di-iron center. The red sphere shows cysteine position. IMM, inner mitochondrial membrane.

As shown in Figure 2A, the protein sequence identity of rTAO vs. rAtAOX1A is 31.04%. However, amino acid residues that comprise the catalytic site are highly conserved, in addition to the iron-binding residues (asterisks marked glutamate and histidine in Figure 2A). Furthermore, it should be noted that TAO contains two cysteine residues (C71 and C95) that are located within the hydrophobic core of the protein (Figure 2B), whereas AtAOX1A contains three cysteines, two of which are located inside the hydrophobic core (C115 and C137 in AtAOX1A) and one on the protein surface (C65 in AtAOX1A). C65 is oriented to the mitochondrial matrix and is the highly conserved cysteine residue found exclusively in the N-terminal domain of plant AOXs (Figure 2B). It has been shown that this residue is the binding site of allosteric activators (such as pyruvate) (Umbach et al., 2006; Selinski et al., 2018). Hence, quinol and oxygen kinetics were compared with or without effectors in the following experiments.

### Comparison of Quinol Kinetics Between rTAO and rAtAOX1A

**Figure 3:**
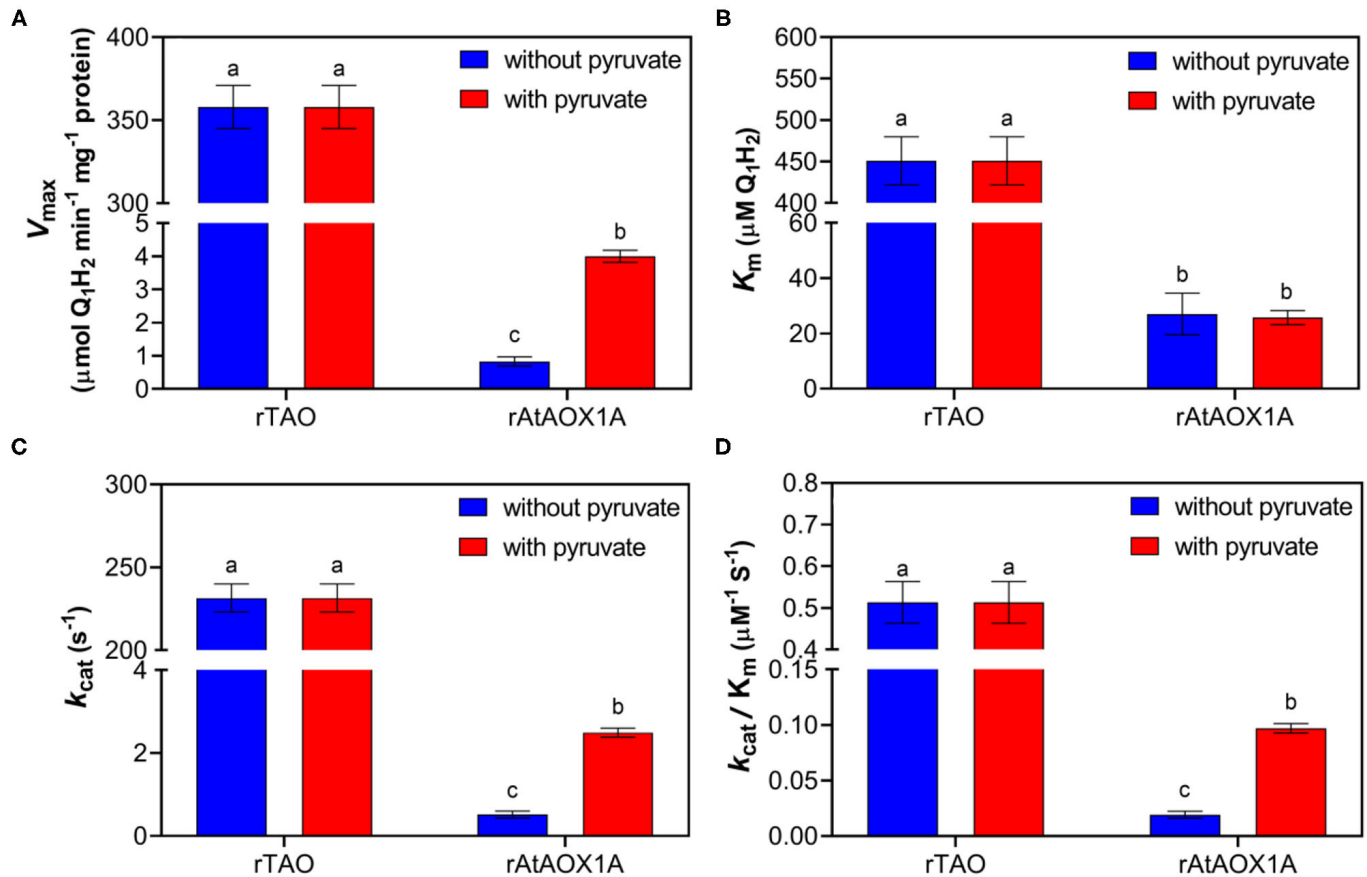
Quinol assay of purified rTAO and rAtAOX1A. The specific activity of purified rTAO and rAtAOX1A were measured spectrophotometrically **(A)**. The kinetic parameters were compared between rTAO and rAtAOX1A, including *K*_m_ **(B)**, *k*_cat_ **(C)**, and *k*_cat_/*K*_m_ **(D)**. Assays were performed in 150 μl MOPS buffer pH 7.5 with different concentrations of quinol-1 as the substrate, with the change in absorbance at 278 nm measured to determine the rate of quinol conversion. In addition, the activities of rTAO and rAtAOX1A with (+) or without (–) 10 mM pyruvate during purification were compared in this experiment. Results are an average of at least three separate preparations. The significant difference *p* < 0.05 was marked as different letters.

To compare the differences in quinol oxidation between rTAO and rAtAOX1A, the purified enzymes were assayed using quinol-1 (Q_1_H_2_) as the substrate to determine kinetic parameters such as *V*_max_ and *K*_m_. Considering that plant AOXs are sensitive to pyruvate, experiments were performed with or without this activator (Figure 3). Our results show that the *V*_max_ of rTAO was 358 μmol Q_1_H_2_·min^−1^·mg^−1^, which was significantly higher than that of rAtAOX1A (4 μmol Q_1_H_2_·min^−1^·mg^−1^). In addition, rTAO displayed the higher *K*_m_ for Q_1_H_2_ (451 μM) when compared with rAtAOX1A (26 μM). The addition of 10 mM pyruvate during the protein purification and in the enzymatic reaction assay changed the kinetics of the rAtAOX1A, that is, the *V*_max_ of rAtAOX1A increased approximately five times, while the *K*_m_ remained unchanged (Figures 3A,B). While this is a significant increase in *V*_max_ when compared with the non-activated form, this is still a significantly lower rate than that determined for TAO.

Further analysis of kinetic parameters showed that rTAO has a higher *k*_cat_ than rAtAOX1A, which was ~90-fold higher than that of rAtAOX1A (Figure 3C). Moreover, the catalytic efficiency (*k*_cat_/*K*_m_ for Q1H2 substrate) of rAtAOX1A was more than 5-fold lower than that of rTAO (Figure 3D). However, pyruvate increased the maximum reaction rate and the efficiency of enzyme catalytic reaction for rAtAOX1A.

### Comparison of Oxygen Consumption Rates Between rTAO and rAtAOX1A

**Figure 4:**
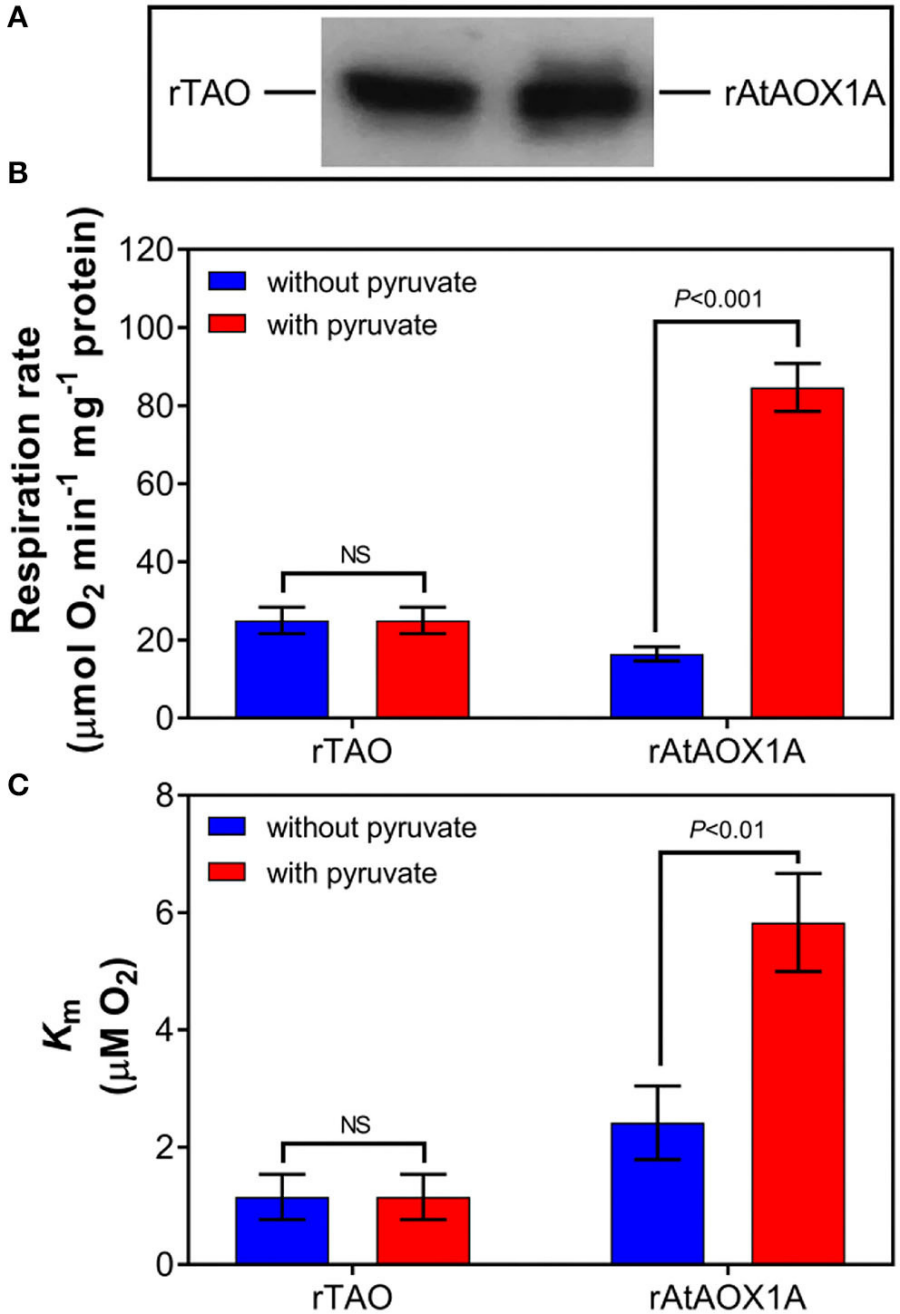
Oxygen kinetic parameters of rTAO and rAtAOX1A. Membrane samples were run in 2 ml of a MOPS buffer pH 7.5 with (+) or without (–) 10 mM pyruvate, with 1.25 mM NADH in an oxygen electrode (OROBOROS instrument). **(A)** Determination of the signal intensity of immunodecorated rTAO and rAtAOX1A by SDS–PAGE after separation of 20 μg of total membrane proteins; the pixel intensities obtained by the Image J software and the relative protein concentrations were calculated based on the signal intensity of immunodecorated purified rTAO and rAtAOX1A. Respiration rate **(B)** and *K*_m_ **(C)** for O_2_ were measured and compared with or without pyruvate. Results are an average of at least three separate preparations. The significant difference *p* < 0.05 was marked as different letters. NS, not statistically significant.

To further compare the kinetic parameters of rTAO and rAtAOX1A, the oxygen consumption rates and *K*_m_ for oxygen were determined with membrane-bound proteins. As shown in Figure 4, the respiratory rate of membrane samples containing rTAO was 25 μmol O_2_ min^−1^ mg^−1^ protein with a *K*_m_ of 1.15 μM O_2_, which is substantially lower than the *K*_m_ for rAtAOX1A. In comparison, the oxygen consumption rate of rAtAOX1A membrane samples was 16.5 μmol O_2_ min^−1^ mg^−1^ protein with a *K*_m_ of 2.41 μM O_2_. When 10 mM pyruvate was introduced into the reaction chamber, it significantly increased the oxygen consumption rates of rAtAOX1A but not that of rTAO. The respiratory rates of rAtAOX1A were ~6-fold higher than those without pyruvate (Figure 4), which is comparable to the increase observed in *V*_max_. Furthermore, the addition of pyruvate resulted in an increased oxygen consumption rate and an increased *K*_m_ value for O_2_ for the membrane bound rAtAOX1A. In short, these results indicate that rTAO has a stronger O_2_ affinity than rAtAOX1A and that pyruvate plays an important role in the acceleration of turnover of the plant AOX (rAtAOX1A).

### Influence of Different Effectors on the Activities of rTAO and rAtAOX1A

**Figure 5:**
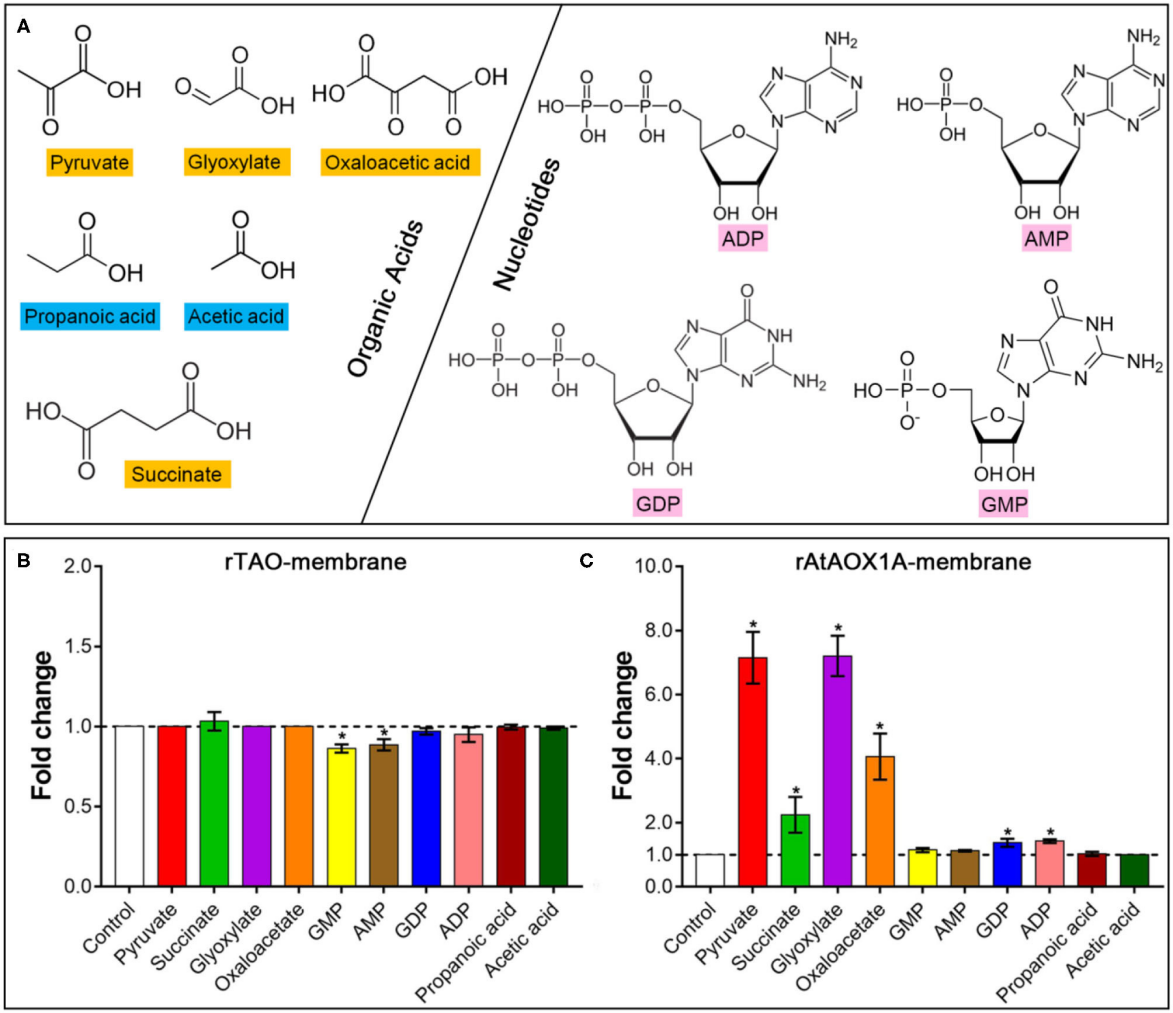
Effects of different effectors on the oxygen consumption rate of AOX proteins. **(A)** Chemical structures of organic acids and nucleotides are shown. Fold changes of rTAO **(B)** and rAtAOX1A **(C)** after treatment with different effectors. Within this experiment, membrane-bound proteins were used for the activation test. For the substrate, 1 mM NADH was used, and 1 mM KCN was added to avoid the leaking respiration pass through complex IV. Basal activities (no effector, control) were ~8 μmol O_2_ min^−1^ mg^−1^ protein for rTAO and rAtAOX1A. According to the results of titration, 10 mM of effectors were used in this experiment. Asterisks (*) represent the significant difference (*p* < 0.05) between the treated samples and the control.

To compare the post-translational regulation by different effectors between rTAO and rAtAOX1A, organic acids, such as succinate, pyruvate, glyoxylate, and oxaloacetate, and nucleotides, such as AMP, GMP, ADP, and GDP, were used in this study (Figure 5A). As shown in Figure 5B, no significant activation was observed by any of these effectors for rTAO. In comparison, it should be noted that succinate, pyruvate, glyoxylate, and oxaloacetate dramatically promoted the oxygen consumption rates of rAtAOX1A, especially for pyruvate and glyoxylate, where a more than 7-fold induction was observed when compared with the control (Figure 5C). In addition, the rAtAOX1A can also be stimulated by both ADP and GDP, although activation was relatively weak, with only a 1.4-fold increase in oxygen consumption. Considering the stronger activation of plant AOXs by pyruvate or glyoxylate, other biologically relevant α-keto acids, such as propanoic acid and acetic acid, were also used to investigate the influence on the oxygen consumption rates of rAtAOX1A (Figure 5). The results showed that there was no stimulation by these two organic acids on the activities of rAtAOX1A, indicating that α-keto acids play important roles in the regulation of rAtAOX1A.

**Figure 6:**
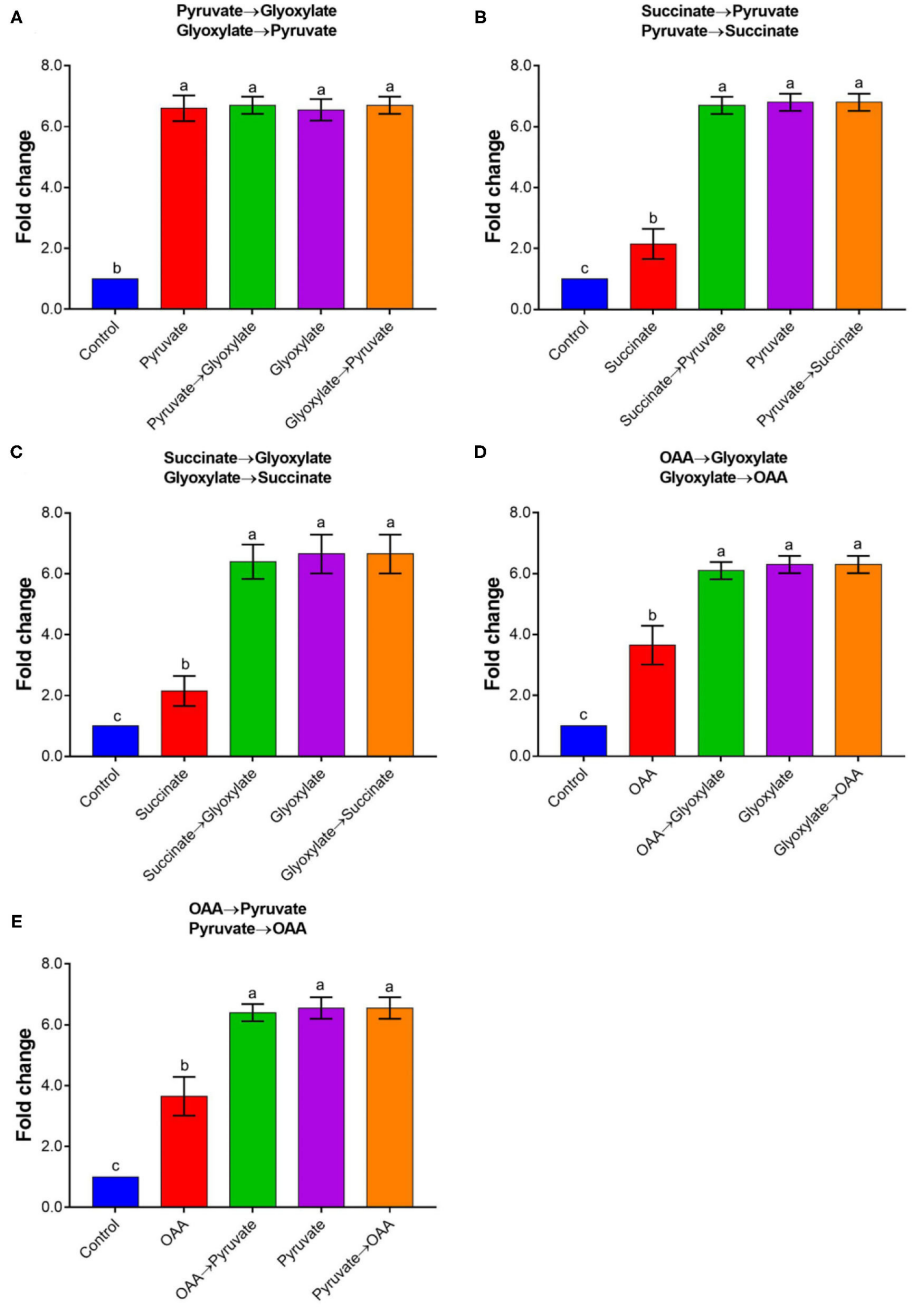
The combined activation of rAtAOX1A by different organic acids. Changes in rAtAOX1A oxygen consumption rate after successive treatments with pyruvate/glyoxylate **(A)**, pyruvate/succinate **(B)**, succinate/glyoxylate **(C)**, OAA/glyoxylate **(D)**, and OAA/pyruvate **(E)**. Within this experiment, the membrane-bound AtAOX1A protein was used for the activation test. For the substrate, 1 mM NADH was used as and 1 mM KCN was added to avoid the leaking respiration pass through complex IV. Basal activities (no effector, control) were ~8 μmol O_2_ min^−1^ mg^−1^ protein for rAtAOX1A, and 10 mM of effectors were used in this experiment. Data are mean values ± SD for five independent experiments. Different letters mark statistically significant differences (*p* < 0.05).

To explore whether the effector has a cumulative effect, different effectors were introduced into the reaction system in sequential order. It should be noted that there was no further activation of rAtAOX1A by adding 10 mM pyruvate followed by 10 mM glyoxylate (Figure 6). Similarly, no further activation occurred by adding 10 mM glyoxylate followed by 10 mM pyruvate. Interestingly, the results showed that 10 mM pyruvate and glyoxylate could further improve the activities of rAtAOX1A if it is stimulated by succinate or oxaloacetate. In contrast, succinate and oxaloacetate did not further stimulate the activities of rAtAOX1A when they were previously activated by pyruvate or glyoxylate. Moreover, a similar increase in fold changes was obtained when 10 mM pyruvate or 10 mM glyoxylate was added into the measurement, indicating that pyruvate and glyoxylate, but not other α-keto acids, are the key effectors that contribute to the full activation of rAtAOX1A.

### Comparison of the Inhibitory Properties Between rTAO and rAtAOX1A

**Figure 7:**
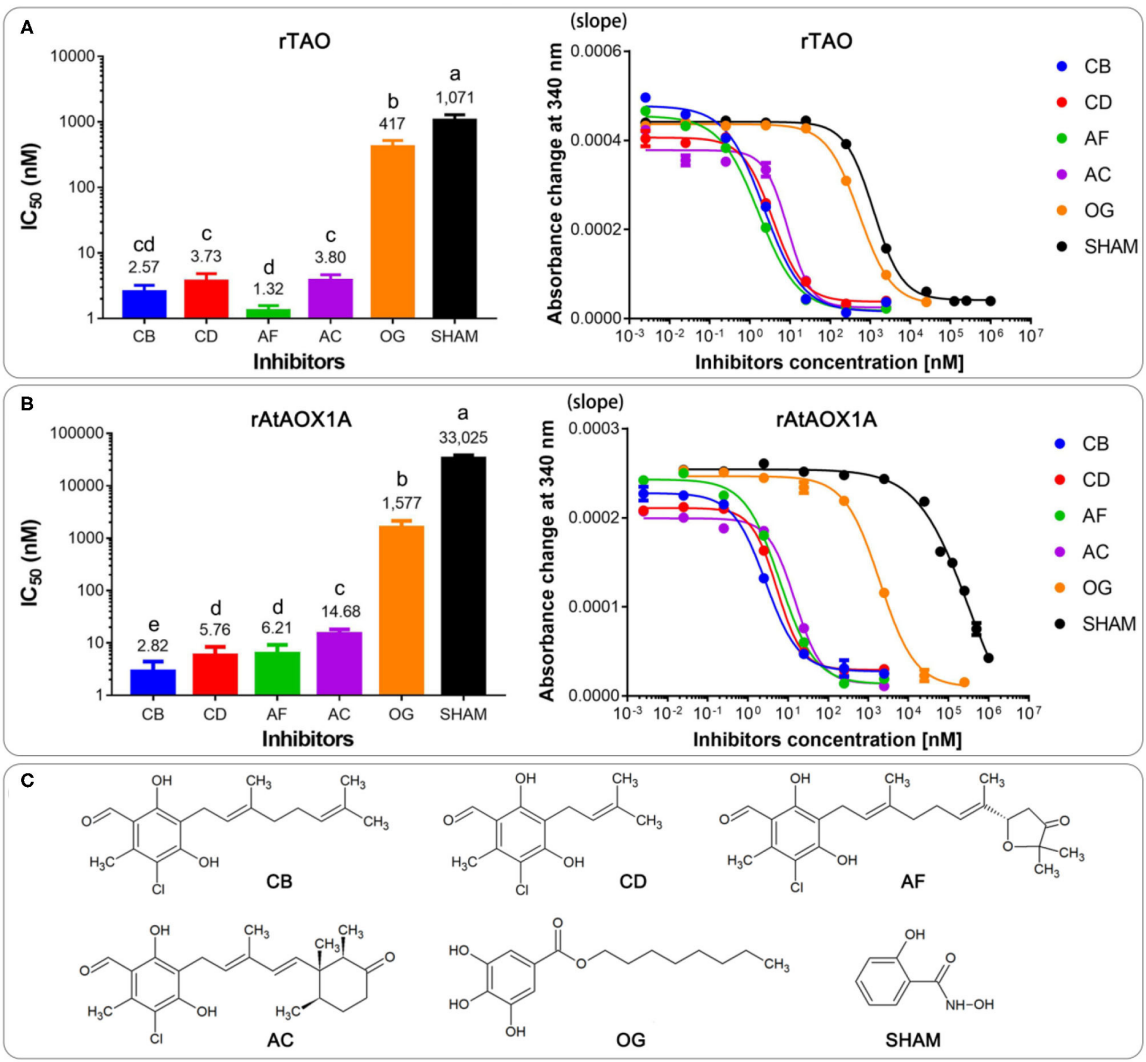
Comparison of IC_50_ values of the membrane-bound proteins of rTAO **(A)** and rAtAOX1A **(B)** with different inhibitors. **(C)** The chemical structures of inhibitors were used in this experiment. Data are mean ± SD of five replicates. For the IC_50_ measurement, absorbance changes of NADH at 340 nm with different inhibitors in a MOPS buffer (pH 7.5) were detected with 300 μM NADH as the substrate. The significant difference *p* < 0.05 was marked as different letters.

Classical inhibitors of the alternative pathway are SHAM and OG (McDonald, 2008). However, AF and its derivatives have been identified as much more potent AOX inhibitors (Saimoto et al., 2013; West et al., 2017). Here, we sought to test SHAM, OG, and four novel inhibitors, namely, AF, AC, CB, and CD, and compared their action on AOXs from different organisms (Figure 7).

Dose-response assays were performed with membrane-bound AOX to determine the IC_50_ value of each compound. Results showed that the most potent rTAO inhibitor is AF (IC_50_ = 1.3 nM), followed by CB (IC_50_ = 2.5 nM), AC (IC_50_ = 3.7 nM), and CD (IC_50_ = 3.8 nM). In comparison to these four inhibitors, OG and SHAM are less potent inhibitors against rTAO, especially SHAM, whose IC_50_ was >1 μM (Figure 7A). Notably, it seems that CB is the best inhibitor against rAtAOX1A, with IC_50_ values of 2.8 nM. For rAtAOX1A, CD and AF showed similar IC_50_ values, which were higher than the value of CB and lower than the value of AC. The inhibition of AC was much less potent than CB, CD, and AF when it was applied to rAtAOX1A, but as still much more effective than OG and SHAM against this protein. The results showed that IC_50_ values of 14.7 nM were detected when AC was used to inhibit the activity of rAtAOX1A (Figure 7B). As with rTAO, the traditional inhibitors OG and SHAM were less effective against rAtAOX1A compared with the AF derivatives, although OG was better than SHAM. It should be noted that OG and SHAM are more powerful inhibitors against parasitic AOX (rTAO) than the plant AOX (rAtAOX1A), especially SHAM, where the IC_50_ values were more than 20 times higher for the rAtAOX1A than for rTAO.

### Comparison of Homology Protein Structures of TAO and AtAOX1A

**Figure 8:**
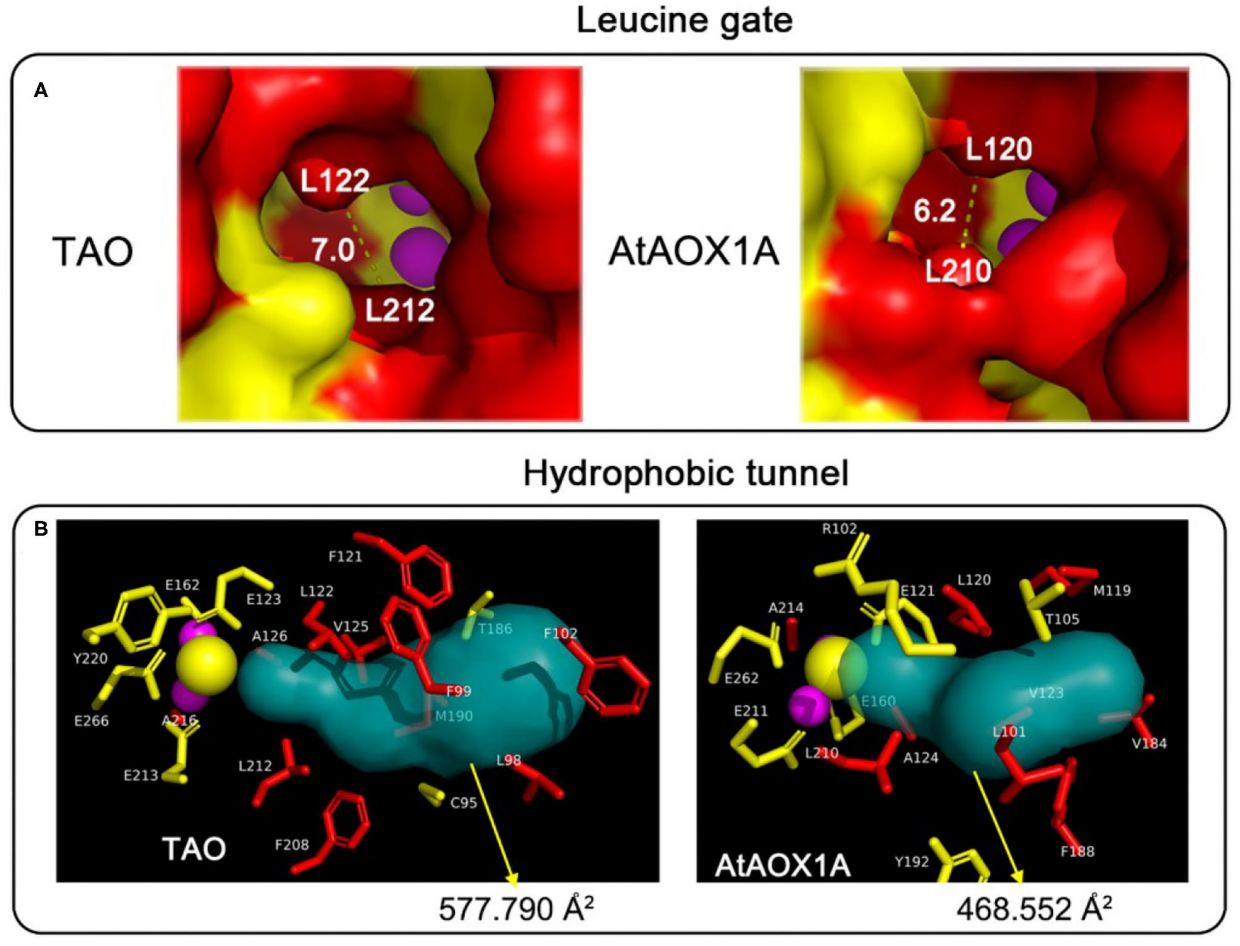
Comparison of the protein tunnel between TAO and AtAOX1A. **(A)** Comparison of the leucine gate between TAO and AtAOX1A. Schematic representation of the entrance to the ubiquinone/ubiquinol cavity for TAO and AtAOX1A, respectively. The red-colored surface represented the hydrophobic cavity, whereas the yellow-colored surface represented the hydrophilic residues. **(B)** Comparison of the quinol tunnel and the atoms around the quinol tunnel between TAO and AtAOX1A. The hydrophobic tunnels were analyzed using Caver 3.0 software. Atoms around the hydrophobic tunnel within 6 Å were analyzed, and the side chain of these residues was shown in a different color. Red, hydrophobic residues; yellow, hydrophilic residues; purple sphere, di-iron center; yellow sphere, OH^−^.

To learn more about the mechanism of the difference in protein activity between TAO and AtAOX1A, the homology protein structures, especially the quinol tunnel, were analyzed. As shown in Figure 8A, the leucine gate, which is proposed to direct ubiquinol (and analogous inhibitors) into the active site by allowing the head group to “dog-leg” toward the di-iron core (Young et al., 2013), forms a bottleneck of ~6 Å in AtAOX1A, while it is ~7 Å in TAO. Moreover, a Caver analysis showed that TAO has a larger hydrophobic cavity leading from the membrane side of the protein to the di-iron core than that of AtAOX1A. In comparison, the surface area of the hydrophobic tunnel is ~577 Å^2^ in TAO, while it is 468 Å^2^ in AtAOX1A (Figure 8B). When the residues in the hydrophobic tunnel are compared, it is apparent that highly conserved hydrophobic residues constitute the backbone of the cavities in both (Figure 8B; Table 1). However, AtAOX1A contains phenylalanine (F188) instead of a methionine (M190) in TAO that is located close to the entrance of the cavity, which, in our opinion, results in a narrower channel for AtAOX1A, thereby affecting the affinity and orientation of the substrate and inhibitor prior to their entrance into the active site (Figure 8B; Table 1).

**Table 1:** Residues lining the cavity of TAO and AtAOX1A.

**Proteins**	**Hydrophobic residues**
TAO	**L98**, F102, F121, **L122**, **V125**, **A126**, M190, **L212**, F208, **A216**
AtAOX1A	**L101**, M119, **L120**, **V123**, **A124**, V184, F188, **L210**, **A214**

*Residues around the hydrophobic tunnel within 6 Å were compared and the bold fonts are the conserved residues surrounding the tunnel*.

## Discussion

In this study, we described and compared the kinetic parameters of AOXs from two different organisms, namely, a human parasite (*T. brucei*) and a model plant (*A. thaliana*). Although the substrate-binding pocket and the catalytic amino acid residues are highly conserved between the two enzymes, we observed a range of values for *K*_m_ and *V*_max_ for quinol-1 and *K*_m_ for oxygen, with rTAO exhibiting much higher *V*_max_ and *K*_m_ for quinol-1 but a lower *K*_m_ for oxygen than that of rAtAOX1A. It is generally accepted that the bloodstream form of *T. brucei* relies exclusively on TAO for respiration during host infection (Chaudhuri et al., 2006) and, given this dependency, substrate kinetics would need to be considerably faster than in homeostatic systems given the crucial nature of the process. Although such a role may explain why TAO has a stronger affinity for oxygen, it does not explain why TAO has a higher *K*_m_ for quinol (more than 300 μM) among all published AOXs (Kido et al., 2010; Young et al., 2020). Nevertheless, even with a lower affinity for quinol, rTAO still possesses a greater catalytic efficiency (*k*_cat_/*K*_m_) than that of rAtAOX1A in the present study.

It is well established that plant AOXs are stimulated by carboxylic acids, typically pyruvate (Millar et al., 1993; Siedow and Umbach, 2000; Umbach et al., 2006; Selinski et al., 2017). Here, we confirmed that the rAtAOX1A is similarly more sensitive to pyruvate and glyoxylate. It appears that the addition of pyruvate into both protein purification and kinetic assay buffers facilitates the purification of the fully active form of rAtAOX1A. This is in agreement with results obtained with *A. maculatum* AOX (Carre et al., 2011), *A. thaliana* (Selinski et al., 2017), and *S. guttatum* (Elliott et al., 2014), suggesting that a general protocol for plant AOX purification should include pyruvate to guarantee maximal enzymatic activity. Strikingly, the activity of rTAO is apparently higher than rAtAOX1A regardless of the addition of activators, suggesting that rTAO is generally in its fully active form, which fits with its role as the sole terminal oxidase. As lower protists, the AOXs from *Euglena gracilis, Paramecium tetraurelia*, and *Acanthamoeba karinii* do not show the same fully active state as *T. brucei* but is activated by purine nucleotides (Sharpless and Butow, 1970; Doussière and Vignais, 1984; Woyda-Ploszczyca et al., 2009). This may also be due to the fact that *T. brucei* is parasitic in animals, and it is less disturbed by environmental conditions compared with other protists or plants. For Arabidopsis AOX1A, pyruvate and glyoxylate can fully activate the enzyme based on the data in this study and previous reports (Selinski et al., 2018), possibly due to the avoidance of reduced or inactivated forms of a rAtAOX1A dimer. By adding different effectors successively, the results in this report demonstrate that, if pyruvate and glyoxylate are added first, other effectors do not further enhance the activity of rAtAOX1A. On the other hand, if other effectors such as succinate and OAA are added first, then pyruvate and glyoxylate can further enhance the activity of rAtAOX1A, indicating that the structure of the keto-acid is important for activation. Such a notion is supported by the result that, when pyruvate and glyoxylate derivatives were used as effectors, the activation of AOX was significantly reduced.

Interestingly, this study showed that pyruvate increased the rate of O_2_ consumption but appeared to decrease the affinity of AtAOX1A for O_2_ (the increased *K*_m_ for O_2_). In fact, the integrated relationship between the affinity of AOX for O_2_ and its maximum O_2_ consumption rate seems to have caused previous confusion. It has been found that, when the *V*_max_ of AOX decreases, the *K*_m_ value also decreases (this seems to increase the affinity of AOX for O_2_) (Young et al., 2014). Conversely, when the *V*_max_ of AOX increases, it is accompanied by an increase in *K*_m_ (this seems to decrease the affinity of AOX for O_2_). We have suggested that the steady-state level of at least one O_2_-derived AOX intermediate will increase when the *V*_max_ of AOX decreases (Crichton et al., 2010). We speculated that, in this study, the increase in the O_2_ consumption rate of AtAOX1A when pyruvate is added may be due to secondary structure rearrangement, which reduces the steady-state level of O_2_-derived AOX intermediates but accelerates the operation of O_2_. Moreover, Fersht (1999) states that the maximization of rates requires high values of *K*_m_. One reason is that a low *K*_m_, which means good substrate binding, results in an energetic pit, hence, more energy is required to move the reaction on to the highest energy state (the transition state). Ideally, an enzyme binds the transition state better than the unchanged substrate. In other words, if the activator decreases substrate binding (higher *K*_m_) without distorting transition state binding, it would be expected that *V*_max_ increases. However, further research is required to determine the underlying reason for the decrease in O_2_ affinity.

In addition to differences in catalytic efficiency, there were significant differences in inhibitor sensitivity between rTAO and rAtAOX1A. Although AF was the most potent inhibitor against rTAO, this compound exhibited a higher IC_50_ in plant AOXs and was even worse than CB for rAtAOX1A. Moreover, AC displayed the same patterns as AF, i.e., it is less effective against plant AOX (rAtAOX1A) than protozoan AOX (rTAO). Overall, such results indicate that CB and CD are broad-spectrum AOX inhibitors, while AF and AC are slightly more selective toward TAO. A comparison of their chemical structures reveals that those four compounds have identical pharmacophores but different tail substituents (Young et al., 2020; Rosell-Hidalgo et al., 2021). While CB and CD have pure isoprenoid tails, AC and AF contain substituted six-atom and five-atom rings at the end of the isoprenoid tail, respectively, which might restrict interaction with the protein ligand-binding site in plant AOXs due to a narrower entrance into the hydrophobic cavity than calculated for TAO (May et al., 2017). Nevertheless, it is obvious that conventional inhibitors such as OG and SHAM are less potent than the novel inhibitors used in this study, which is consistent with previous reports on *Sauromatum* (Elliott et al., 2014; May et al., 2017).

The differing kinetic properties of AOXs might also be attributed to the variable size of the hydrophobic cavity with different isoforms even though the residues surrounding the catalytic center are essentially conserved (Figures 2, 8). Based on the structure of TAO, we analyzed the homology modeling of AtAOX1A, and the residues lining the quinol tunnel were compared. It is conceivable that the larger hydrophobic cavity calculated for TAO should prove beneficial for substrate egress into the catalytic center. A further factor to consider is the presence of the leucine gate within the hydrophobic cavity (May et al., 2017). In AtAOX1A, the smaller aperture of this gate in comparison to that calculated for TAO may further restrict substrate entry into the active site, thereby decreasing its catalytic efficiency. It should be noted that, when we compared the hydrophobic tunnel of TAO and AtAOX1A, although there is significant homology between the two species, phenylalanine (F188) observed in AtAOX1A is closer to the entrance of the cavity than the smaller methionine (M190) seen with TAO. It is plausible that the positioning of such a large residue may affect the passage of the substrate or inhibitor through unwanted steric hindrance, particularly with respect to the isoprene tail of the compounds. Such a proposal may explain why the reduction in inhibitory efficacy of AC was lower than that of CB, CD, and AF for AtAOX1A in comparison to TAO. Obviously, in order to support such a suggestion, further research is necessary to confirm this idea, with simple point mutations in an otherwise innocuous part of the protein likely to be valuable.

## Data Availability Statement

The original contributions presented in the study are included in the article/supplementary material, further inquiries can be directed to the corresponding author.

## Dedication

We would like to dedicate this article to the memory of my great friend and colleague James Nash Siedow. His pioneering research on the structure and function of the AOX in plants and fungi has provided the impetus for worldwide research on this topic. His great enthusiasm for AOX and the smiles he brought to our faces will always be with us. Thank you for giving me the reason to continue researching this amazing protein!

## Author Contributions

AM: conceptualization, writing review and editing, supervision, and funding acquisition. FX: investigation, data analysis, and writing of the original draft. AC, LY, and MB: investigation, data analysis, and writing review and editing. MA: methodology and writing review and editing. All authors read and approved the manuscript.

## Funding

Research in the laboratory of AM has been supported by the Biotechnology and Biological Research Council (BB/L022915/1 and BB/NO10051/1). We also acknowledge funding from the National Natural Science Foundation of China (31400242 and 31900242).

## Conflict of Interest

AM, LY, and MA declare they have financial interests in AlternOx Scientific Ltd. The remaining authors declare that the research was conducted in the absence of any commercial or financial relationships that could be construed as a potential conflict of interest.

## Publisher's Note

All claims expressed in this article are solely those of the authors and do not necessarily represent those of their affiliated organizations, or those of the publisher, the editors and the reviewers. Any product that may be evaluated in this article, or claim that may be made by its manufacturer, is not guaranteed or endorsed by the publisher.
